# Tribological Investigations on Tool Surfaces for Temperature-Supported Forming of Magnesium AZ31 Sheets

**DOI:** 10.3390/ma13112465

**Published:** 2020-05-27

**Authors:** Bruno Caetano dos Santos Silva, Alisson Mendes Rodrigues, Roland Mueller, Fábio André Lora, Marcio Luis Ferreira Nascimento, Haroldo Cavalcanti Pinto, Rodrigo Santiago Coelho

**Affiliations:** 1 SENAI CIMATEC, Institute of Innovation for Forming and Joining of Materials, Av. Orlando Gomes, 1845, Piatã, Salvador-BA 41650-010, Brazil; bruno.silva@fieb.org.br; 2 Materials Engineering Unit (UAEMa), Federal University of Campina Grande (UFCG), Av. Aprígio Veloso, no. 882, Bodocongó, Campina Grande-PB 58109-970, Brazil; alisson_mendes@ymail.com; 3 Department of Materials Engineering-SMM, São Carlos School of Engineering–EESC, University of São Paulo–USP, São Carlos-SP 05508-010, Brazil; haroldo@sc.usp.br; 4 Fraunhofer Institute for Machine Tools and Forming Technology (IWU), Reichenhainer Str. 88, D09126 Chemnitz, Germany; roland.mueller@iwu.fraunhofer.de; 5 Materials Department, Federal University of Recôncavo Baiano–UFRB, Av. Centenário, 697, Feira de Santana-BA 44085-132, Brazil; fabio.lora@ufrb.edu.br; 6 Nanotechnology Group, Graduate Program in Industrial Engineering, Polytechnic School, Federal University of Bahia, Rua Aristides Novis 2, Federação, Salvador-BA 40210-630, Brazil; mlfn@ufba.br

**Keywords:** forming process, tool design, magnesium alloy AZ31, periodic surface structures, friction coefficient

## Abstract

Aiming to decrease friction coefficient (μ) during the forming of magnesium alloy sheets, nine (9) tools with different hole geometries in their surface (flat, elliptical, and circular) were manufactured from steel Boehler W400 VMR (as known as DIN 1.2343). Tribological investigations were accomplished on a strip drawing machine at 288 °C without lubricants. When compared with a standard tool (surface flat), on average, tools with circular geometries in their surface showed the smallest friction coefficient, while tools with elliptical geometries shown higher. The friction coefficient also was confronted with the ratio between area occupied by holes in the surface of the tool and the total tool surface (i.e., factor *f* (%)), hole diameter (Ø), and the distance between circle centers (d(c,c)). Principal Component Analysis (PCA) complemented the experimental approach. In summary, both approaches (experimental and theoretical) indicated that the manufactured tool with circular geometries on its surface presented lower friction coefficient values on the forming processes of the magnesium AZ31 sheets.

## 1. Introduction

Energy efficiency increasing by means of less fuel consumption is directly related to metal forming processes. Researches on these technologies are essential for the automotive industry to accomplish the vehicle weight reduction. Magnesium has been demonstrated as a material with significant potential for weight reduction due to its low density (1.74 g/cm³) and relatively high mechanical strength. However, the use of lubricants and the relatively high values of the friction coefficient are the biggest problems to forming sheets from magnesium alloys [1,2,3].

Magnesium alloys have a limited forming capacity at room temperature [3,4], which is associated with their hexagonal closed packed structure [5]. Thus, it is necessary to enable additional gliding planes, which can be obtained by performing the processes at temperatures above 225 °C, in order to produce components of complex geometries [3]. Notwithstanding, when the forming temperature exceeds 230 °C, the lubrication choice is more restricted to mineral oil, grease, molybdenum disulfide, a colloidal graphite solution, fiberglass, as well as Teflon [6]. After the forming process, these lubricants should be removed from magnesium alloys parts, as soon as possible, in order to prevent corrosion and problems in their cleaning [7,8,9]. Additionally, if the lubricant cannot be removed from the component surface, the subsequent layer applied to prevent corrosion will present poor adhesion.

The friction is an important phenomenon that intervenes in almost all manufacturing processes, arising in the interface between the materials [10,11]. In the sheet metal forming process, the friction is influenced by parameters, such as material properties, surface finish, temperature, sliding velocity, contact pressure, and lubricant characteristics [9,12].

The magnitude and distribution of friction affect the metal flow, part defects, and quality, as well as production costs [7,9,13]. In deep drawing operations, lubricants are used to enable the workpiece to slide under the blank holder area and over tool surface under reduced friction conditions. Minimum surface marking and the possibility to form with a more uniform stretch are some of the benefits of using lubricants [14,15].

Specific tests have been used for simulating industrial forming conditions, including the strip drawing [16], which is a kind of Bending Under Tension Test (BUT). These tests evaluate the friction behavior during blank sliding into a die [17], as well as the resulting tribological conditions [18]. It is also a variation of this test, where the sheet slides between two parallel dies and it is possible to measure the frictional force in that region. Similar experiments can be found in the literature [16,19], where satisfactory results were obtained regarding the friction coefficient during forming.

An alternative to avoiding the post-metal forming process using lubricants consists of developing blank holders with a surface structure specially designed to provide lower friction levels (between the blank holder and the workpiece) during magnesium alloys forming processes. Researches show that surface structures generally consist of cavities that are produced on the forming tool surface by machining processes. Dimples were chosen as shape elements due to their non-directional behavior [20]. A step before manufacturing, the blank holder consists of designing surface structured test tools with the potential of decreasing the friction levels in the forming process and performing friction tests with these tools, aiming to evaluate the friction behavior of each one.

This paper discusses the BUT performed tests in order to determine the friction coefficient, without using lubricants, between magnesium AZ31 strips and test tools designed and manufactured with an especially developed surface structure to reduce the friction and the potential for application of these tools. A principal component analysis (PCA) was performed to answer which of three variables, hole diameter (Ø); distance between circle centers (d(c,c)); and, the ratio between area occupied by holes (elliptical or circular) in the surface of the tool and the total tool surface (factor *f*), is more relevant when considering eight of nine tools used in this work.

## 2. Materials and Methods

**Figure 1:**
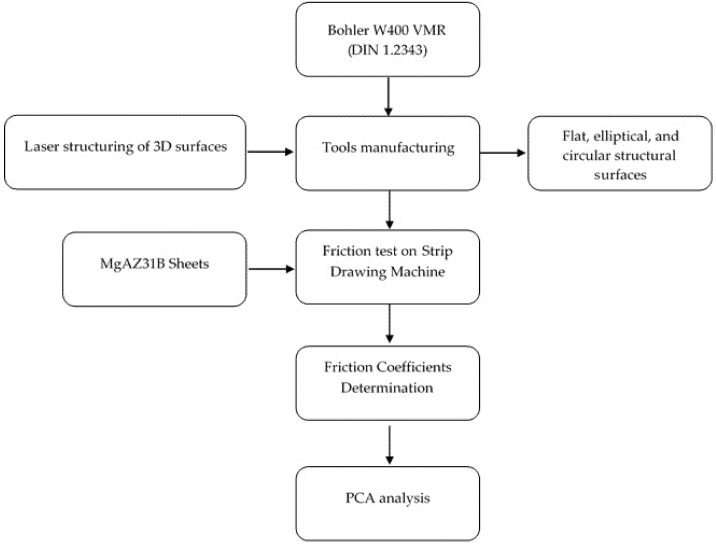
Summarize the experimental and theoretical strategies employed in this work.

Figure 1 summarizes the experimental and theoretical strategies accomplished to investigate the friction coefficient between the manufactured tools and the magnesium AZ31 strips. It is possible to see all of the work stages applied in this study. Starting from the tool manufacturing (the chosen material, cut technology, and the disposal holes in the surface), the tribological tests to determine the friction coefficients until the PCA analysis. Related to the experimental strategy, two main methodological aspects considered corroborate for the further scaling up of the investigated technology: first, the tests were carried out while considering the friction conditions in the tool radius, which represents the most critical condition in the sheet metal forming processes. Secondly, the use of parameters, such as temperature and drawing speed, as compared with the corresponding values used in industrial processes.

### 2.1. Development of the Tools to Friction Tests

**Figure 2:**
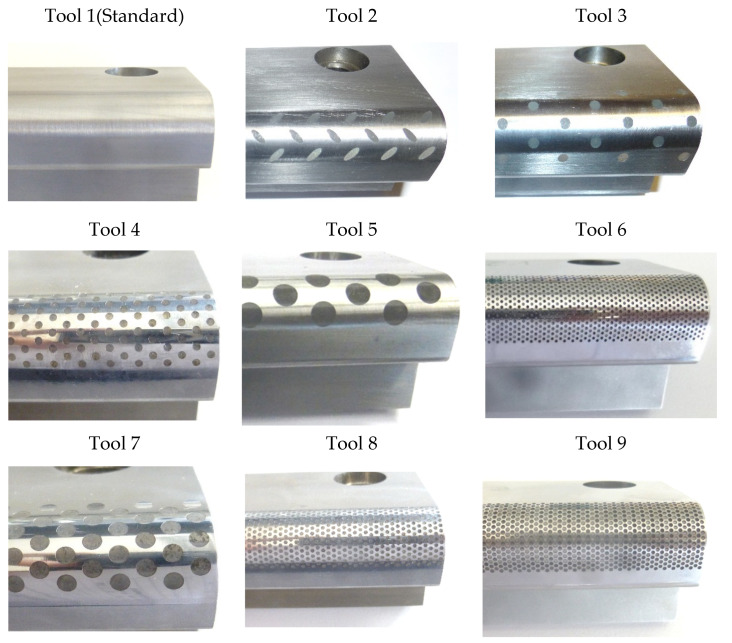
Manufactured tools with different surface structures (flat, elliptical, and circular) development for friction tests.

To tribological investigations, besides the standard tool with a flat surface, more eight tools with elliptical and circular geometries on their surface were manufactured, see Figure 2. Every tool surface was drawn in order to enable a reduction of friction levels between magnesium AZ31 strips and the tools. The surface of the manufactured tools was characterized in terms of the hole diameter (Ø), the distance between circle centers (d(c,c)), and factor *f*, which is defined as the ratio between area occupied by holes (elliptical or circular) in the surface of the tool and the total tool surface, see Table 1. The higher the factor *f* value means, the smaller the contact area between the tool surfaces and the magnesium alloys sheets.

All tools were manufactured using Bohler W400 VMR (as known as DIN 1.2343) at Fraunhofer IWU, Chemnitz, Germany, with a hardness of 52 HRC. This material is characterized by usual excellent wear and hot wear resistance when compared to standard hot working steels [21]. The macrostructure of the tools was finished with a picosecond laser system with 532 nm wavelength and pulse duration of 12 ps. This laser system has a multi-axis arrangement for the laser structuring of three-dimensional (3D) surfaces and an optical measurement system for depth control. The dimples of all surfaces have a depth of 50 µm.

### 2.2. Friction Tests

**Figure 3:**
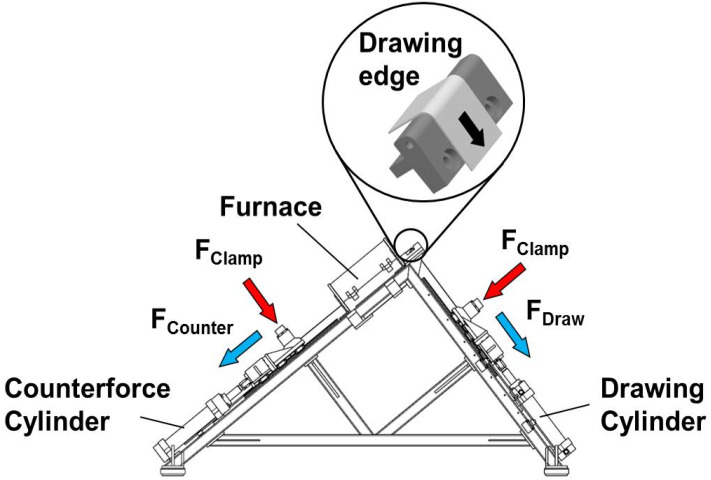
Scheme of Strip Drawing Machine developed at Fraunhofer IWU.

A test model was set up in simulation of the process using the strip drawing equipment developed at Fraunhofer IWU (Chemnitz, Germany) in order to evaluate the friction behavior of the manufactured tools (Figure 3). In highlight in Figure 3, it is shown the test setup with the drawing edge geometry (10 mm radius), which represents the tools in this experiment.

Table 2 illustrates the test parameters for the projected strip drawing test. The length of the furnace is 618 mm. The magnesium AZ31 strips were adapted to have a final length of 2070 mm, of which only 615 mm max. was heated into a furnace. The dwell time for each strip in the furnace before the drawing was 10 min. Subsequently, the strip was manually pushed as far as the drawing edge, the ends were tensioned, and drawn over the drawing edge at a rate of 50 mm/s. The experiments were performed in three trials, using three magnesium AZ31 strips (2.0 mm thickness) for each tool in the first trial and five strips in the second and third trials. Moreover, in the subsequent trial, both the flat test tool (standard) as the tool that performed better results previously were retested. Thus, the number of strips tested for some tools is not the same (Table 3).

The basis for calculating the friction value is the Euler–Eytelwein Rope Friction formula. Based on this rule, the friction value (*µ*) might be derived in accordance with Equation (1), where F_Draw_ is the drawing force (that “pulls” the strip), *F_Counter_* is the counterforce, *F_Bend_* is the pure bending force and $\frac{2}{\pi }$ refers to the 90° angle between the drawing force and counterforce:

(1) $$\mu =\frac{2}{\pi }\ln\frac{F_{Draw}-F_{Bend}}{F_{Counter}}$$

A strip of the metal material to be examined is drawn over a rotating roller (assumed to be mounted in non-friction bearings), as a result of which only bending and not friction forces occur in relation to the roller, in order to be able to calculate the proportion of the bending of the metal strip on the drawing force. The force path is derived from the mean value based on three of the sheets drawn over the bending roller. 

Strip drawing tests were conducted with a 90° bending operation in order to ensure good metrological accessibility and also to illustrates the real forming conditions. When compared to the pin-on-disc test method and the conventional strip drawing, the applied BUT provides corresponding surface contact conditions for sheet metal forming [22,23]. A pump drives the drawing cylinder after starting the test, causing a tractive force on the strip and making it slide over the test tool. During the relative movement between the strip and the tool, a load cell system measures the forces, and the displacement of the cylinder is measured by a system of specified displacement sensors to attend the drawing distance. All of the data are stored on the equipment’s computer, and a spreadsheet is generated for post-analysis. 

The friction values determined for each tool development are applied to a temperature range of approx. 235–260 °C, which is the temperature of the magnesium strip at the beginning of the drawing, out of the furnace.

### 2.3. Fundamentals the Principal Component Analysis (PCA)

In brief, what PCA does is to determine new variables, termed principal components (PC’s), which account for the majority of the variability in the data. The instructions for calculating PC’s are somewhat simple. The first PC is the direction throughout the data that explains the highest variability. The second and subsequent PCs must be orthogonal to the previous PC and describe the maximum amount of the remaining variability [24]. The same analysis can be done, while considering correlation instead of variance [25].

The principal component analysis is a singular case of transforming the original data into a new coordinate system. If the original data involve *n* diverse variables, then each datum may be considered as a point in an *n*-dimensional vector space. From a practical point of view, data from Table 1 could be represented by a matrix R composed of *n* = 3 variables (Ø, *d*(*c*,*c*) and *f*) and *t* = 8 objects (one was discarded, specifically about the tool 2, due to lack of data). A normalization is proposed to proceed with further calculations, changing original matrix R into standard matrix X.

PCA is, in simple terms, a rotation of the system of original axes into another one that attempts simultaneously: (*i*) to find in the space of dimension *n* a specific direction in which data spread at the maximum form and in which it is expected to find a pattern; (*ii*) to protect the maximum original information from this method. This is done while using finding a covariance matrix XX^T^, where X^T^ is the transpose matrix of X (i.e., the normalized data). Briefly, a variance is a “measure of data spread” when considering a unique variable. Covariance is similar to variance, but it considers data from different variables. From this procedure, the eigenvectors represent the cosine directors (or the contribution that each one of the original axes gives to the composition of the new axes), named main components. The eigenvalues, in turn, correspond the amount of original variance for the respective eigenvectors.

#### Matrix Algebra Derivation of the Principal Component Analysis

The mathematics of the matrix approach is given below. Let the data be represented as a matrix R with *t* rows and *n* columns, where *n* is the number of variables and *t* is the number of observations (or lessons). It is relevant to consider the raw data transformed by a normalized matrix X. Every *X_i_* can be described as:

(2) $$X_{i}=\overset{t}{\underset{i=1}{\sum }}\frac{R_{i}-{\bar{R}}_{i}}{\sqrt{Var\left(R_{i},R_{i}\right)}}$$

where ${\bar{R}}_{i}$ corresponds to average (or mean) of variable *i* and Var(*R_i_*,*R_i_*) is the variance or a “measure of data spread” while considering a unique variable *X_i_*, 1 < *i* < *t*:

(3) $$\mathrm{Var}(R_{i},R_{i})=\sum_{i=1}^{t}\frac{{\left(R_{i}-{\bar{R}}_{i}\right)}^{2}}{t-1}.$$

The matrix of approximations of the data based upon the vector of coefficients A and the vector of the principal component P is obtained by multiplying the *n*×1 vector A times the 1×*t* vector, which is the transpose of the vector P; i.e.,

AP^T^. (4)

The matrix of deviations of the actual data from the values-based upon A and P is:

X−AP^T^. (5)

The sum of the squared deviations can be obtained in terms of matrix operations by multiplying the deviations matrix times the transpose of the deviation matrix itself; i.e.:

(X − AP^T^)(X − AP^T^)^T^. (6)

The result is a *n* × *n* matrix. The sum of the elements on the principal diagonal is the sum of the squared deviations for all of the variables. The constraint on the choice of A is that:

A^T^A = I. (7)

The first-order condition for a minimum concerning the elements of P is, in matrix form:

P^T^ = A^T^X (8)

or, equivalently,

P = X^T^A. (9)

The first-order condition for a minimum with respect to the elements of A is, in matrix form:

λA^T^ = XP, (10)

or, correspondingly, since P = X^T^A,

λA = XX^T^A. (11)

where λ is the eigenvalue. Letting B = AA^T^ (the covariance matrix), named:

λA = B^T^Y. (12)

Thus, A is an eigenvector of the matrix B. The equation defining eigenvalues and eigenvectors is:

(B − λI)A = 0,(13)

where I is an identity matrix, and the zero on the right side represents a vector of zeroes. For this equation to have a nontrivial solution, the determinant of the coefficient matrix (B−λI) must be zero. This is the condition determining the eigenvalues that are the variances themselves. This procedure reduces to an *n*-th degree polynomial equation. Once an eigenvalue λ*_k_* (1 < *k* < *n*) is obtained, the system of equations can be solved for the components of A by setting one component equal to an arbitrary nonzero value and then solving for the rest. The solution can be normalized and put it into any desired form. The eigenvalues, in turn, correspond the amount of original variance for the respective eigenvectors, following the order of relevance related to every PC: λ_1_ > λ_2_ > … > λ*_n_* and with Σλ*_k_* = *n*. Equations (4)–(13) summarize the PCA standard procedure.

## 3. Results

**Figure 4:**
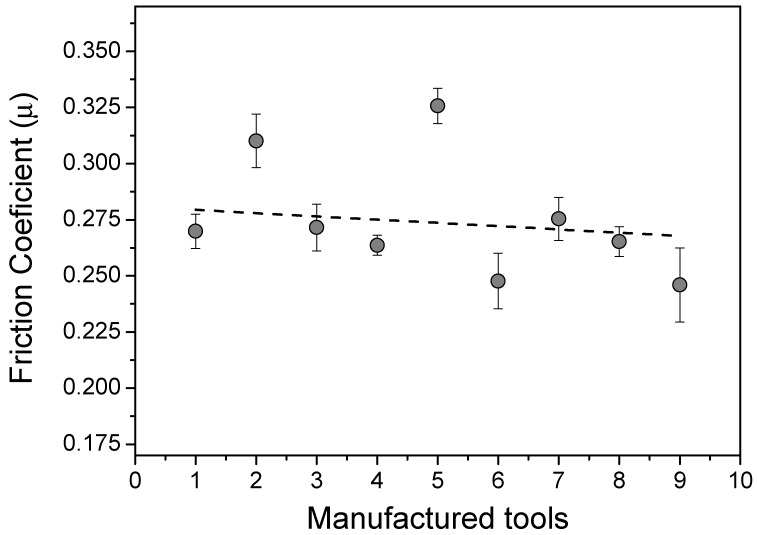
Comparison between the friction coefficients and each manufactured tool in this work.

Figure 4 shows the mean friction coefficient (*µ*) measured between the magnesium AZ31 strips and the different tools that were developed in this work. The experimental values were determined after 100 mm of running test in order to ensure stable behavior of the Strip Drawing Machine. Mousavi et al. [26] found similar results related to the numerical simulation of steel sheet forming using tools with a superficial structure and slightly inferior to the results obtained by Wakuda et al. [27]. The last evaluating the surface structure effect on the friction reduction in the contact between ceramic and steel. However, the latter used lubrication in the contact area. Taltavull et al. [28] evaluated the tribological behavior of the AM50B magnesium alloy sliding without lubrication against carbon steel discs without structured surface while using a pin-on-disc tribometer and obtained friction coefficient values around 0.25, for loads that were applied above 40 N. Similar tribological studies between magnesium alloy sheets and structured surface tools were not found in the literature.

**Figure 5:**
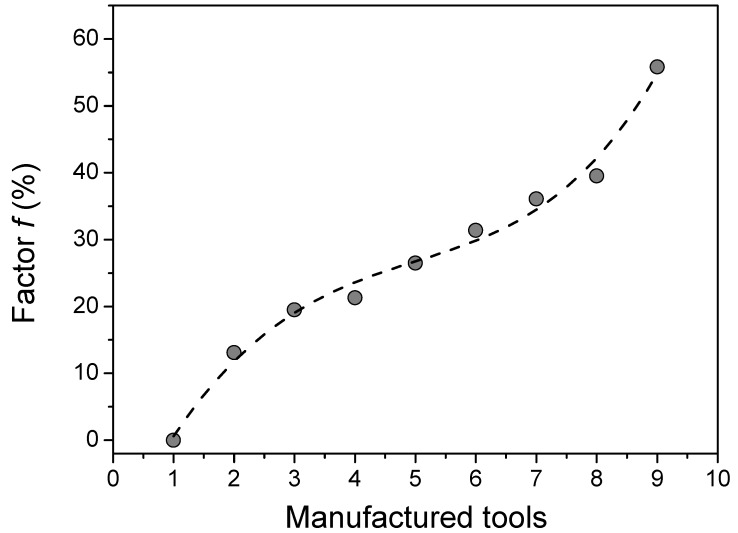
The plot of factor *f* (%) for the manufactured tools in this work.

Figure 5 shows a plot of factor *f* (%) for the tools. In Figure 5, it was observed a very interesting trend, i.e., the factor *f* increased using different tools. The higher the *f* value means, the smaller the contact area between the tool surfaces and the magnesium alloys sheets. The smaller contact area reduces the shear stresses that are caused by the frictional forces [26,29,30]. Based on this, this paper focused on increasing the *f* values of the tools to investigate the respective frictional behavior. However, there are two other relevant parameters, the hole diameter (Ø) and the distance between circle centers (d(c,c)), to take into account. From data available, it was possible to find some statistical correlations between Ø, d(c,c), tools, and the f factor using Principal Component Analysis (PCA).

## 4. Discussion

It can be seen that the friction coefficient (*µ*) showed smooth differences for the nine test tools, according to the results that were acquired from the experimental test. The flat surface tool (standard) has presented *µ* = 0.270. The smallest friction condition was founded on tool 9, with *μ* = 0.246, a reduction of 8.9% in comparison with the standard tool. The highest value was found for the tool 5 (*µ* = 0.326), representing an increase of 20.7% also in relation to the standard tool.

Analyzing Figure 4, it is possible to note that the average friction coefficients presents a relatively high standard deviation. This is likely related to the strip material adhesion on the tools surface, which impacts increasing the surface roughness, leading to a variation in the measured friction coefficient for the subsequent strip. For example, it was noted an increase in the friction coefficient from first to the second strip in almost all tools. The tool 2 (elliptical) and 5 (Ø_5_ = 6.00 mm/d_5_(c,c) = 10.00 mm) presented overly high *μ* values, behaving as outliers, see Figure 4. For the elliptical geometry, it is presumed that the ellipses inclination in relation to the strip drawing direction acted as a barrier to the material slip, consequently increasing the friction. For tool 5, it is supposed that due to the large diameter of the holes, the strip was tapping the base of the hole, increasing the contact with the tool, and hindering the material sliding. Although the objective in this work has been to reproduce industrial manufacturing conditions, the high standard deviation measured to the friction coefficients can be considered to be a limitation of the applied approach.

**Figure 6:**
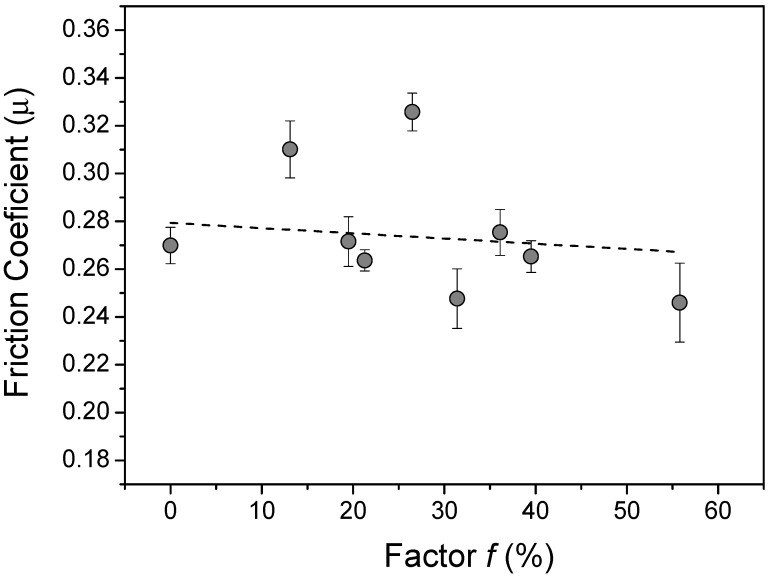
The relation between *f* (%) and *µ* for all manufactured tools in this work.

An expected tendency was the reduction of the friction coefficient by increasing the factor *f*. However, not all the tools followed this trend, as can be seen in Figure 6. For example, tools 2 and 5 (considered outliers) presented an increase in *µ* by increasing the *f* value. Additionally, tools 6 and 9 did not follow this expected trend because presented lower values of the diameter of the holes and distance between circle centers, which contributed to a decrease in the value of the friction coefficient. Despite these outlier’s experimental data, tools 1, 3, 4, 7 and 8 have presented lower *µ* value by increasing the *f* values.

When comparing tools 3 and 4, the parameters (Ø) and d(c,c) were reduced by half, see Table 1. These changes have impacted the factor *f* significantly. As a result, smaller *μ* experimental values were measured for test tools with higher factor *f*. A similar phenomenon was observed between test tools 7 and 8, except that in this situation, the parameters were reduced by one third.

In relation to the test tools 6 and 9, it was kept the d(c,c) = 1.25 mm, and the holes diameter was varied, Ø_6_ = 0.75 mm and Ø_9_ = 1.0 mm. These changes led to a higher value of *f* for the tool 9 (Table 1) and a reduction of 0.8% in *μ*. Another analysis was performed among tools 8 and 9, which both have the same holes diameter (Ø = 1.0 mm) and different distance between centers (d_8_(c,c) = 1.5 mm and d_9_(c,c) = 1.25 mm): the changes resulted in an increase in factor *f* for the test tool 9, and a reduction of 7.2% in *μ* was observed.

**Figure 7:**
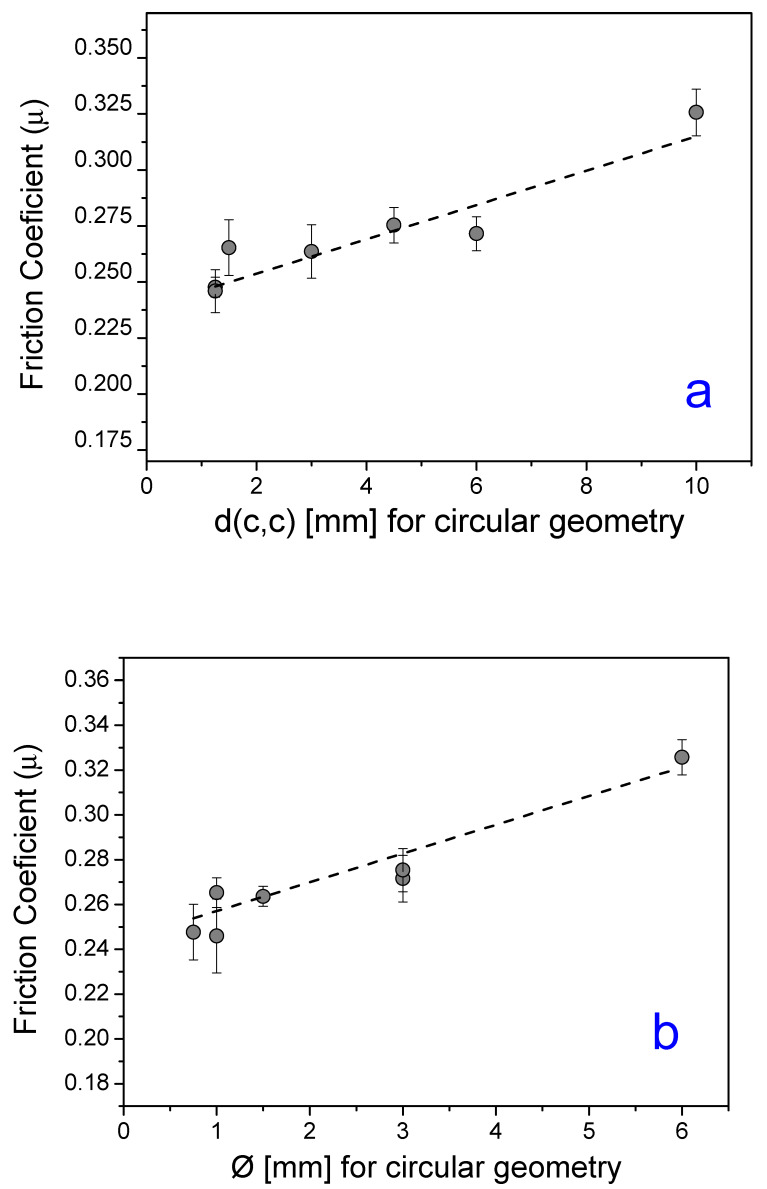
(**a**) Relation between d(c,c) and *µ*; (**b**) Relation between Ø and *µ*; both for manufactured tools with circular structure surface geometry.

Based on the analysis mentioned above, it was observed that, for tools with a circular surface structure, in general, both the increase in the hole diameters (Ø) as in the distance between the circumferences centers d(c,c), individually, led to an increased friction coefficient, as can be seen in Figure 7a,b. It is believed that, due to the large diameter of the cavities, with the application of the load, the contact between the strip and the base of these cavities was quite expressive. The roughness of this region is superior to the surface roughness of the tool, since it was not subjected to the polishing process, which made it difficult for the material to slip, generating an increase in friction in the process, as previously mentioned.

Regarding PCA, the results have shown that it is possible to correlate the three geometric parameters: hole diameter (Ø), the distance between circle centers (d(c,c), and the *f* factor. 

Table 1 presents the data that were used for the PCA procedure. High variance corresponds to high distance d(c,c) values ranging from 0 mm to 6 mm, according to the PCA theory. By definition, d(c,c) values correspond to the first PC and the second one to factor *f*. From Table 4 98.99% of the data could be represented by the first two axes (related to distance d(c,c), representing 52.17% of variability, and factor *f*, representing 46.82% of variability, respectively). Positive eigenvalues occurred due to the use of medium values from the original data. Table 5 shows the centered correlation matrix corresponding to Table 1.

Table 4 shows the total and cumulative percentages and the respective eigenvalues of each axis considered (distance, factor, tools, and diameter, in order). Note that the eigenvalue sum is four, i.e., the number of variables analyzed, in agreement with PCA theory. The first component PC_1_ can be expressed in terms of normalized *X_i_* variables and corresponding values of the correlation matrix as:

PC_1_ = − 0.491*X*_1_ + 0.513*X*_2_ + 0.554*X*_3_ − 0.434*X*_4_ (14)

The coefficients of the first principal component in Equation (14) are related to tools, diameter, distance, and factor *f*, and their respective eigenvectors. That is to say, PC_1_ will be high if *X*_2_ and *X*_3_ are high, with a predominance of the last. Thus, the difference in coefficients shows that 52.17% of the variation in the data is related to distance. The negative coefficients of *X*_1_ and *X*_4_ (related to tools and factor *f*) mean that the value of these variables affects PC_1_, decreasing it.

The second principal component can be similarly interpreted, with a predominance of factor *f* due to its highest coefficient:

PC_2_ = + 0.506*X*_1_ + 0.488*X*_2_ + 0.436*X*_3_ + 0.562*X*_4_ (15)

**Figure 8:**
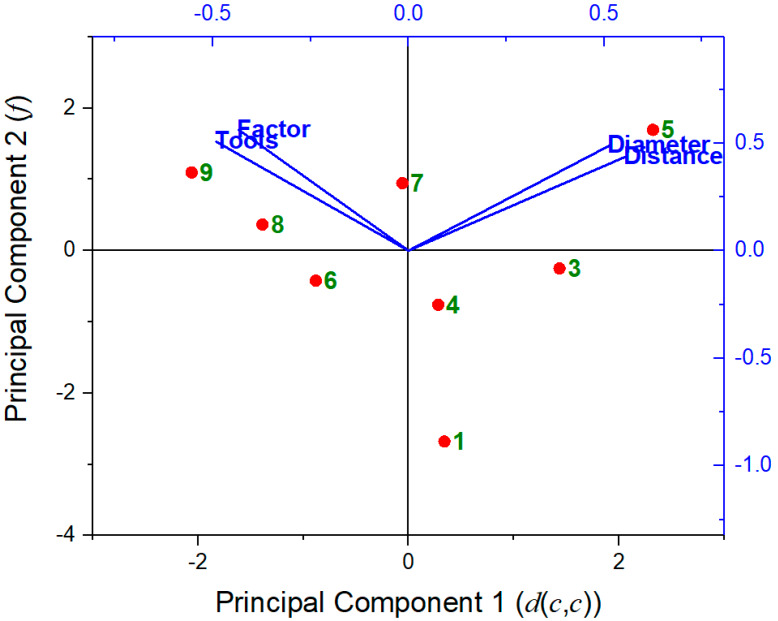
Biplot of principal component 1 (related to distance d(c,c) parameter) versus principal component 2 (factor *f*), considering the correlation mode [13].

Figure 8 shows the mapping distribution of principal component 1 (PC1, related to d(c,c)), and principal component 2 (PC2, related to *f*). This result agrees with the previous analysis that showed the smallest friction condition related to tool 9, with *μ* = 0.246, a reduction of 8.9% in comparison with the standard tool. PCA mapping presented these data near the lowest PC component factor *f*. On the other hand, the highest value was found for the tool 5 (*µ* = 0.326), clear related to first PC’s, the distance d(c,c), the diameter Ø, and the factor *f*.

According to Equation (14), the diameter is also important on PC_1_ due to its correlation with distance:

(16) $$\mathrm{Corr}(X_{i},X_{j})=\frac{\sum_{i=1}^{n}\left(X_{i}-{\bar{X}}_{i}\right)\left(X_{j}-{\bar{X}}_{j}\right)}{\sqrt{Var\left(X_{i},X_{j}\right)}\sqrt{Var\left(X_{i},X_{j}\right)}}$$

for every *i* ≠ *j*. When *i* = *j*, the Corr(*X_i_*,*X_i_*) = Var(*X_i_*,*X_i_*) is 1, by definition.

From Equation (16), it is possible to observe that the correlation coefficient is the covariance of two variables *X_i_* and *X_j_* divided by the product of their squared root variances. Correlation indicates both the strength and direction of the linear relationship between two variables. However, different from covariance is dimensionless [13]. 

The correlation value should lie between −1 and + 1. A coefficient of +1 specifies that the two variables are perfectly positively correlated: as one variable increases, the other also increases by a comparable quantity. However, this does not mean that the variation in one variable causes the other to change, only that their changes coincide. On the other hand, a coefficient of −1 shows a perfect negative relationship: if one variable increases, the other decreases by a comparable amount. A coefficient of zero implies that there is no linear relationship between the variables [13]. 

The results show that diameter and distance parameters are highly correlated, with a coefficient correlation Corr(*X_d_*, *X*_∅_) = 0.989 when considering all data available in Table 5, and in agreement with Equation (16). Another high correlation was observed between tools and factor *f*, with Corr(*X_tools_*, *X_f_*) = 0.975. Such resulting correlations influenced the first calculated PC components, as predicted by theory.

## 5. Conclusions

Experimental tests were accomplished to evaluate the friction behavior between tools with different surfaces (flat, elliptical, and circular) and magnesium AZ31 strips. The tool with circular geometries on its surface (tool 9) showed a reduction of 8.9% on the friction coefficient when compared with a standard tool (tool 1). The same was not observed for tools with ellipsoidal geometries in its surface (tool 2), in which the friction coefficient increased in comparison with the tool 1 (0.270 and 0.310), respectively. This behavior occurred, because, in elliptical geometries, it is presumed that the ellipses inclination in relation to the strip drawing direction acted as a barrier for the material slip. When confronted with factor *f* (%), Ø, and (d(c,c)), the friction coefficient acquired from tools with circular geometries (except tool 5, the best result, also verified by PCA) were lower than those that were obtained from standard and ellipsoidal tolls. Additionally, it was possible to determine the distance d(c,c) as the first principal component, representing 52.17% of variability, and the factor *f* as the second principal component, representing 46.82% of variability, respectively. Therefore, it can be inferred that tools with circular geometries on their surface is an innovative research and they have the potential to represent a solution to reduce the friction in deep drawing processes of magnesium AZ31 sheets. However, this solution still needs to be improved since the lowest friction coefficient that is obtained is still not sufficiently low to make it possible to implement such a forming process without using lubricants. Future researches may consider carrying out detailed analyzes of the adhesion mechanism and evaluate alternatives to remove the material adhered to the test tools surfaces before the subsequent test, ensuring an identical tool surface for all of the tests.

## Figures and Tables

**Table 1:** Surfaces parameters of the manufactured tools.

Tool	1	2	3	4	5	6	7	8	9
Geometry	Flat	Elliptical	Circular						
Ø [mm]	–	A = 2/b = 5	3.00	1.50	6.00	0.75	3.00	1.00	1.00
d(c,c) [mm]	–	7.00	6.00	3.00	10.00	1.25	4.50	1.50	1.25
*f* (%)	–	13.09	19.51	21.30	26.50	31.40	36.10	39.50	55.80

**Table 2:** Strip Drawing Test Parameters.

Parameter	Value
Drawing velocity v_Draw_ [mm/s]:	50
Strip Temperature inside the furnace T_F_* [°C]:	288
Dwell time in furnace t [min]	10
Tool temperature T_T_* [°C]:	245
Tool Surface Roughness (Rz) [µm]:	1.57
Strip width [mm]:	70
Strip Surface Roughness (Rz) [µm]:	10.5–11.4
Drawing distance [mm]:	200
Lubrication:	Without

* three points average temperature at the beginning of the test.

**Table 3:** Number of Magnesium AZ31 Strips Tested for Each Tool.

Tool	1	2	3	4	5	6	7	8	9
N° of Strips	13	3	8	5	3	5	5	10	5

**Table 4:** Total and cumulative percentages and respective eigenvalues of each axis considered.

PC axis	Eigenvalues	Total Percent (%)	Cumulative Percent (%)
1	2.08692	52.17	52.17
2	1.873	46.82	98.99
3	0.03767	0.94	99.93
4	0.00242	0.06	100.00

**Table 5:** Correlation matrix A of data presented in Table 1.

	Tools	Diameter Ø	Distance d	Factor f
Tools	1	−0.05695	−0.1592	0.96039
Diameter Ø	−0.05695	1	0.98907	0.04185
Distance d	−0.1592	0.98907	1	−0.03791
Factor f	0.96039	0.04185	−0.03791	1

## References

[1] Davies G., Davies G. (2012). Chapter 4—The role of demonstration, concept and competition cars. Materials for Automobile Bodies.

[2] Wetzel T. (2012). Magnesiumblech-Technologiekette für Innovative Leichtbauanwendungen im Automobilbau.

[3] Schieck F., Drossel W.G., Bräunlich H., Scheffler S., Pierschel N. (2013). Temperature-supported forming of automobile related magnesium components. Proceedings of the ASME International Mechanical Engineering Congress and Exposition, Proceedings (IMECE).

[4] Song J., She J., Chen D., Pan F. (2020). Latest research advances on magnesium and magnesium alloys worldwide. J. Magnes. Alloy..

[5] Jiang S., Jiang Z., Chen Q. (2019). Deformation twinning mechanism in hexagonal-close-packed crystals. Sci. Rep..

[6] Shi Z., Wang L., Mohamed M., Balint D.S., Lin J., Stanton M., Watson D., Dean T.A. (2017). A new design of friction test rig and determination of friction coefficient when warm forming an aluminium alloy. Procedia Eng..

[7] Avedesian M.M.H.B. (1999). Magnesium and Magnesium Alloys.

[8] Kaya S. (2008). Improving the Formability Limts of Lightweight Metal Alloy Sheet Using Advanced Processes-Finite Element Modeling and Experimental Validation. Ph.D. Thesis.

[9] Totten G.E., Totten G.E. (2017). ASM Handbook, Volume 18: Friction, Lubrication, and Wear Technology.

[10] Suh B.-C., Kim J.H., Hwang J.H., Shim M.-S., Kim N.J. (2016). Twinning-mediated formability in Mg alloys. Sci. Rep..

[11] Ugender S., Kumar A., Reddy A.S. (2014). Microstructure and mechanical properties of AZ31B magnesium alloy by friction stir welding. Procedia Mater. Sci..

[12] Meng Y., Xu J., Jin Z., Prakash B., Hu Y. (2020). A review of recent advances in tribology. Friction.

[13] Fang J.H., Pan F.S., Chen B.S., Wu J., Dong L. (2011). Friction and wear performances of magnesium alloy against steel under lubrication of rapeseed oil with S-containing additive. Trans. Nonferrous Met. Soc. China Engl. Ed..

[14] BlueScopeSteel (2003). Technical Bulletin TB-F1: Lubrification of Stell Sheet and Strip for Forming.

[15] Maruda R.W., Krolczyk G.M., Wojciechowski S., Powalka B., Klos S., Szczotkarz N., Matuszak M., Khanna N. (2020). Evaluation of turning with different cooling-lubricating techniques in terms of surface integrity and tribologic properties. Tribol. Int..

[16] Wichern C.M., Van Tyne C.J. (1999). Frictional behavior of the sliding interface between an A2 steel die and zinc-coated steel sheet. J. Mater. Eng. Perform..

[17] Wiklund D., Rosén B.-G., Wihlborg A. (2009). A friction model evaluated with results from a bending-under-tension test. Tribol. Int..

[18] Vollertsen F., Hu Z. (2006). Tribological size effects in sheet metal forming measured by a strip drawing test. Cirp Ann..

[19] Lee B.H., Keum Y.T., Wagoner R.H. (2002). Modeling of the friction caused by lubrication and surface roughness in sheet metal forming. J. Mater. Process. Technol..

[20] Börner R., Scholz P., Kühn R., Schubert A., Zeidler H., Müller R., Leach R. (2015). Micro structuring of coated tools for dry sheet metal forming of aluminium alloys. Conference Proceedings of the 15th International Conference of the European Society for Precision Engineering and Nanotechnology (EUSPEN).

[21] BÖHLER. W400 VMR Data Sheets^®^. Technical Report, BÖHLER Edelstahl GmbH & Co KG, Kapfenberg, Austria, 2013. https://www.bohleredelstahl.com/.

[22] Müller R., Mosel A. (2014). Characterisation of tool coatings for press hardening. Adv. Mater. Res..

[23] Schieck F., Hochmuth C., Polster S., Mosel A. (2011). Modern tool design for component grading incorporating simulation models, efficient tool cooling concepts and tool coating systems. CIRP J. Manuf. Sci. Technol..

[24] Luis Ferreira Nascimento M., Aparicio C. (2007). Viscosity of strong and fragile glass-forming liquids investigated by means of principal component analysis. J. Phys. Chem. Solids.

[25] Flury B. (1997). A First Course in Multivariate Statistics.

[26] Mousavi A., Schomäcker M., Brosius A. (2014). Macro and micro structuring of deep drawing’s tools for lubricant free forming. Procedia Eng..

[27] Wakuda M., Yamauchi Y., Kanzaki S., Yasuda Y. (2003). Effect of surface texturing on friction reduction between ceramic and steel materials under lubricated sliding contact. Wear.

[28] Taltavull C., Shi Z., Torres B., Rams J., Atrens A. (2014). Influence of the chloride ion concentration on the corrosion of high-purity Mg, ZE41 and AZ91 in buffered Hank’s solution. J. Mater. Sci. Mater. Med..

[29] Kim D.E., Cha K.H., Sung I.H., Bryan J. (2002). Design of surface micro-structures for friction control in micro-systems applications. CIRP Ann. Manuf. Technol..

[30] Karbasian H., Tekkaya A.E. (2010). A review on hot stamping. J. Mater. Process. Technol..

